# Bilateral Neovascular Glaucoma With Retinitis Pigmentosa: A Clinical Challenge

**DOI:** 10.7759/cureus.25915

**Published:** 2022-06-13

**Authors:** Rakhi D Cruz, Aparna Rao

**Affiliations:** 1 Glaucoma, L V Prasad Eye Institute, Mithu Tulsi Chanrai Campus, Bhubaneswar, IND

**Keywords:** retinal ischemia, refractory glaucoma, rubeosis iridis, neovascular glaucoma, retinitis pigmentosa

## Abstract

Retinitis pigmentosa (RP) and glaucoma are frequent associations. Masking of the underlying retinal ischemia is known to be caused by RP that may cause a clinical dilemma and treatment. We present a middle-aged healthy male presenting with bilateral refractory neovascular glaucoma (NVG) and classical RP with no evidence of posterior segment ischemia. The case highlights important points and tailored investigations to arrive at the final diagnosis and treatment for such challenging cases.

## Introduction

Neovascular glaucoma (NVG) is commonly associated with retinal vascular occlusions, proliferative diabetic retinopathy (PDR), and ocular ischemic syndrome (OIS). Retinitis pigmentosa (RP) is an ocular condition characterized by photoreceptor degeneration and reduced retinal oxygen requirements that are known to confer a protective effect against the development of proliferative retinopathy [1-3]. It is believed that reduced demand by photoreceptor degeneration in RP reduces the ischemic load of the retina thereby masking ischemic retinopathies [1,3,4]. While classical RP may be associated with open or closed-angle glaucoma, its association with NVG is rare and may cause clinical challenges. The differentials of bilateral NVG in such a case and the challenges in arriving at the final diagnosis in such scenarios are discussed.

## Case presentation

**Figure 1:**
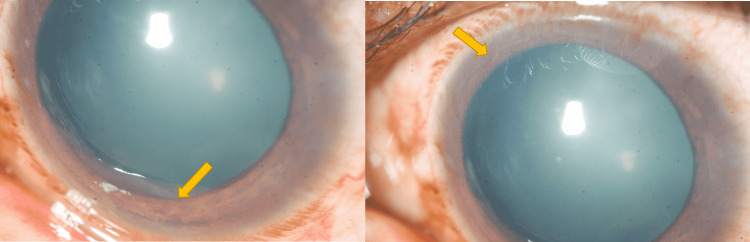
Diffuse slit-lamp microscopic picture of right and left eye showing florid neovascularization of iris with dilated pupils.

**Figure 2:**
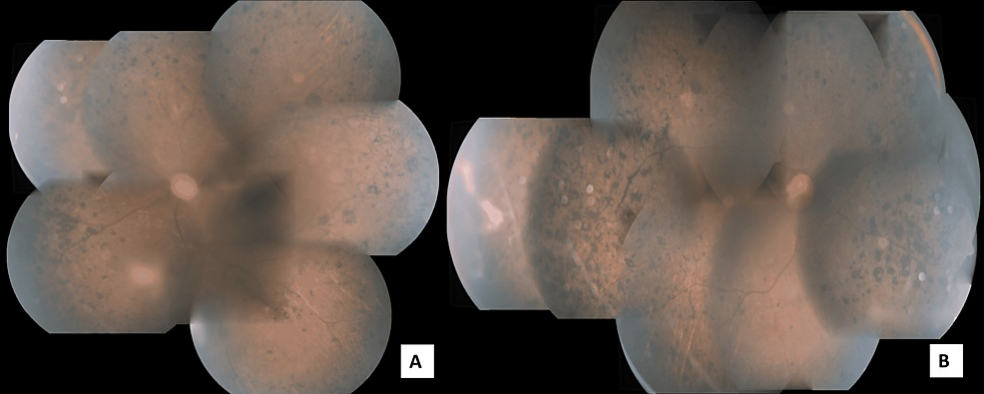
Fundus montage pictures of right and left eye showing the presence of bony spicules, pallor of disc suggestive of RP along with glaucomatous cupping RP: retinitis pigmentosa

A 43-year-old healthy male presented with complaints of sudden onset of pain, photophobia, and watering from both eyes (BE) for 2 weeks. He also gave a history of gradually progressive defective vision over the past 5 years. He also gave a history of defective night vision though there was no positive family history of the same. He was a known diabetic on irregular treatment for 6 months. His best-corrected visual acuity (BCVA) was 20/600 in the right eye (RE) and 20/800 in the left eye (LE). The slit-lamp evaluation showed conjunctival and circumcorneal congestion with microcystic corneal edema, shallow anterior chamber (Van-Herick grade 2), and presence of 360-degree florid neovascularization of iris (NVI) in BE (Figures 1A-1B). The pupil was round, regular, mid-dilated, and non-reacting to light in BE and the lens was cataractous. Gonioscopy revealed scattered peripheral anterior synechiae (PAS) with neovascularization of the angle (NVA). Intraocular pressure by Goldmann-applanation-tonometry (GAT) measured 48 mmHg in RE and 50 mmHg in LE. He was started on maximal medical therapy with low-potent steroids and cycloplegics. Fundus evaluation after resolution of corneal edema revealed features of bilateral RP along with advanced glaucomatous cupping with disc pallor in BE (Figure 2). He was referred to retina services where the absence of diabetic retinopathy or features of surface neovascularization in the retina were noted after peripheral screening (Figures 2A-2B).

Fundus fluorescein angiography was carried out and it revealed no delay in arm-retina and arteriovenous transit time, with no capillary non-perfusion areas ruling out OIS. Complete systemic evaluation with a local physician ruled out other systemic inflammatory and atherosclerotic causes like (coronary-artery diseases, leukemia, carotid occlusive diseases), and collagen vascular diseases (systemic lupus erythematosus, giant cell arteritis, Takayasu arteritis). Bilateral carotid artery doppler revealed normal study with no luminal stenosis or evidence of atherosclerotic plaques. His blood sugar levels, lipid profile, and glycated hemoglobin levels (5.8%) were normal. All renal and other hematological parameters were within normal limits.

The absence of any ischemic etiology in the retina with evident RP pointed toward a diagnosis of neglected primary glaucoma leading to NVG. Though the source of retinal ischemia was not detectable, he was given a trial of injections of intravitreal anti- vascular endothelial growth factor (VEGF) agents with partial pan-retinal photocoagulation (PRP) in the inferior quadrant to reduce any trigger for neovascularization. Uncontrolled intraocular pressure (IOP) despite best efforts mandated trabeculectomy with mitomycin-C (MMC) + intraocular lens surgery along with intracameral bevacizumab for LE. The patient was irregular in postoperative visits and presented at 3 weeks with a localized flat bleb in the LE suggesting failure. Bleb needling with MMC injection (0.004%) helped control the IOP in the LE which gained a vision of 20/100 with evident bilateral optic atrophy. He was referred to the rehabilitation department for mobility and vocational training.

## Discussion

There are few anecdotal reports of the coexistence of retinal neovascularization and RP with some reporting NVG [1-7]. Most of these had some evidence of posterior segment ischemia which was absent in our case that led to the clinical dilemma. The reported mechanisms of NVG in these studies include loss of receptor cells, intraocular inflammation secondary to photoreceptor destruction, co-existent vaso-occlusive diseases, or altered metabolic environment of the retinal vasculature, retinal pigment epithelium (RPE) dysfunction, and capillary non-perfusion [5-8]. Chronically raised IOP by undiagnosed glaucoma was the trigger causing ocular ischemia rather than RP/retinal pathology that explained the failure of any response with retinal PRP in this case. Though this was not mandated, the retina services felt this may reduce the ischemic demand especially if retinal ischemia was being masked by RP and when no other triggers for ischemia were apparent. The absence of retinal neovascularization in the presence of retinal ischemia is well known in RP [2-4]. This led to confusion in the final diagnosis in this case.

Uliss et al. mentioned four cases of RP with neovascularization of which one had NVG with RP with a history of multiple ocular surgeries and other findings like vitreous hemorrhage, subretinal exudation, and diffuse posterior pole edema [3]. Details of systemic inflammatory or ischemic conditions were however lacking in this case. A similar case with inverse RP, cataract, scleromalacia, and NVG has been reported, where no source of neovascularization in the posterior segment was found [4]. The authors hypothesized the cause to be chronic scleritis leading to scleromalacia and ischemia thereby causing NVG.

Differentials for bilateral NVG which were considered in this case included systemic inflammatory diseases/atherosclerotic diseases, carotid occlusive diseases leading to OIS, and PDR masked by RP, all of which were ruled out in our case [3,6-8]. Other causes for isolated rubeosis iridis include ocular tumors, chronic retinal detachment or endophthalmitis, chronic inflammation or uveitis, and ocular trauma [8].

Masking of the underlying retinal ischemia in DR can be masked by RP that can be confirmed by angiography and disc leakage [2]. Our patient’s glycated hemoglobin levels were within normal limits and with no vascular changes suggestive of diabetic retinopathy on angiography. We hypothesized the reason for no evidence of retinal neovascularization to be relatively predominant anterior segment ischemia along with some coexisting retinal ischemia which may have been masked by RP, which prevented the development of evident neovascularization in the posterior segment.

## Conclusions

In conclusion, our cases showed the following insights into NVG in RP. Though a routine differentials diagnosis for bilateral NVG with RP should include OIS, systemic inflammatory diseases like giant cell arteritis causing retinal vascular occlusions, systemic atherosclerotic diseases, masked PDR, neglected asymptomatic chronic glaucoma should also be kept in mind, especially in the absence of evident systemic factors or retinal neovascularization/angiographic abnormalities. Advanced presentation and optic disc damage may cause visual loss which mandates prompt diagnosis and appropriate treatment in such cases.
